# Editorial

**Published:** 1841-03-20

**Authors:** 


### THE MEDICAL EXAMINER.

PHILADELPHIA, MARCH 20, 1841.

### EPIDEMICS OF THE SEASON.

For some weeks the past the prevailing epidemic at Philadelphia has been influenza; as is usually the case, the chief sufferers are children and aged persons, especially the former. In many of them the disease appears as a simple bronchitis, without offering very peculiar symptoms; but in other cases it is extremely severe, and is attended with pneumonia in various degrees of intensity. The pneumonia is in general of the lobular kind, and either succeeds a bronchitis of severe duration, or it occurs more insidiously, as a slow congestion, which does not at first produce much irritation of the air-cells. In some cases the internal congestion is so severe, that reaction establishes itself slowly, and even with difficulty.

The treatment of the disease is not in general very difficult; we have found it necessary to take blood in the smaller proportion of cases, and have relied mainly on mild, *protracted* revulsion, and on ipecacuanha, given so as to cause some nausea, either in the form of wine or syrup. In the more inflammatory cases, when bloodletting was advisable, we have substituted small portions of calomel and antimony for the ipecacuanha. In the declining stages of the affection, when the secretion has become abundant, the lac assafœtidæ, with the addition of a little syrup of senega, is most beneficial.

In the use of revulsives in the inflammations of the chest, especially as they occur in children, it has appeared to us that a mistaken notion is very prevalent, and that the disease is treated by too powerful remedies, which confine their action to a small surface, and irritate the nerves more than they unload the congested vessels. We are much more favourably disposed to milder, but more extended revulsives, which may be applied to a large surface, and allowed to remain in contact with the skin for a long period. These remedies are stimulating foot-baths, repeated several times daily, and poultices to the feet and ankles, made with thick slices of bread moistened with hot vinegar, and dusted with mustard. Over the chest a poultice of onions, made either with simple water, or vinegar and water, acts extremely well.

These are common, every day remedies, but they are just what are wanted, and certainly produce a better and generally a more decided impression upon the disease, than highly stimulating applications, which are in general unsuited to children. What is perhaps still more important is, that in this disease as in many others, the natural tendency is decidedly to a cure; and the nice adaptation of very simple remedies will favour this result, while the less accurate administration of more powerful ones will be almost sure to disturb the natural progress of the disorder.

Another peculiarity has attended this epidemic: some cases of typhus have occurred in the Blockley hospital, and one or two have been admitted there from the city. Those which occurred in the hospital, with a single exception, or at most two, came from one part of the house where the inmates were in constant communication together, but were in a great degree, secluded from the rest of the building; an assistant nurse in the same department also fell ill of the disorder. The disease is in general of a mild type, and offers but little difficulty as to the treatment. It is the proper petechial typhus, attended with the characteristic eruption, and not the typhoid fever. The whole number of cases is fourteen or fifteen, according to the admission or rejection of one case which was admitted very late in the disorder. They are all either doing well or recovered, with a single exception. This patient laboured under phthisis, and was attacked with the fever, which passed through its stages regularly,and after its termination the patient sank in part from his original disease, and in part from the feebleness induced by the attack of fever. As the disorder is of that moderate type which tends naturally to a cure, our treatment has been either laxatives of oil for several days, and the abstinence from any pharmaceutic remedies, or the spirit of mindererus, and other mild diaphoretics. Sinapisms to the extremities, and cold applications to the head, were occasionally prescribed, but the local symptoms were so mild that it was scarcely necessary to resort to local blood-letting in a single case.

The only point of treatment requiring much attention is the necessity of stimulation in most cases towards the close of the disease, and in some at earlier periods. The action of the heart and arteries was feeble, and of course afforded a very certain guide as to the propriety of giving stimulants; when the feebleness occurred late in the disorder, it generally accompanied the decline of the febrile excitement, and sometimes occurred very suddenly. The prostration requires more attention than any other symptom connected with the present epidemic; and when patients fall into it, the only safeguard is the use of wine and other excitants of a similar kind.

---

Every reader of medical literature must be struck with the infrequency of unsuccessful cases recorded. Of late years, indeed since the increased cultivation of pathology, we we have derived much valuable statistical matter from the *medical* wards of many of our hospitals, where the cases presented have been faithfully noted and communicated without selection or suppression. That there is, however, a general ndisposition to the publication of cases terminating unsuccessfully, will be evident to any one who will turn over the pages of a series of medical periodicals. Even of the Alms House Reports, and Hospital cases, the greater portion are palpably served up to illustrate the successful issue of a favourite remedy or mode of treatment; while of cases selected from private practice, does any reader remember as many as he can count on his fingers where diagnosis is represented as faulty or the therapeutics inefficacious? How many surgeons, too, report the operations in which they have failed? In fact, do not the pages of our journals shine forth a brilliant record of triumphant art, very different from the real results of our bed-side experience? It may be thought injudicious to hazard these remarks; they may be liable to misconstruction or perversion, but we think the true interests of science will be advanced by proclaiming the truth and calling attention to the fault. It has not been our practice nor is it our present aim to assume a censorious tone. But as members of a common profession, with the same interests, and certainly no exemption from the same failings as those we address, we claim the right to comment on what strikes us as a prevailing tendency to a pernicious error. In our editorial career, we have seen suppressed the history of more than one most interesting epidemic, carefully prepared for publication, because of the mortality that accompanied it; we have had more than one promise of the record of an operation, *provided* it should be successful—we have time and again seen most honorable and distinguished men, having really at heart the advancement of their profession, and assured of their own position, shrink from the publication of fatal cases, though fatal in defiance of whatever skill and attention could effect.

It is unnecessary to attempt at length the illustration of the point we wish to urge. It is a point generally conceded *if*, not as generally acted upon. We dwell upon it without pretension to advance any thing not generally felt, but from a belief that the mere stirring up of the subject will be advantageous. We of course mean no personal allusion.

---

We have been requested by Dr. Warrington to insert the following correction to his paper on the Cæsarian section, published in our last number. With this we cheerfully comply, as an act of simple justice to the distinguished Professor in whose hands the operations alluded to were followed by such complete success. The cases were reported by Drs. Nancrede and Fox, and the author inadvertently referred to them as *their* cases. The operations were performed by Dr. Gibson.

*Erratum.*—In last line of the first paragraph of the second column of page 167 of the last number of the Examiner, instead of the word "of," read *reported by*, before the words Nancrede and Fox.

The Cæsarean section was performed on Mrs. R——, the subject of the reports alluded to, by Professor Gibson, of the University of Pennsylvania.

